# Five-year monitoring trends in physical performance among junior tennis players

**DOI:** 10.3389/fspor.2026.1890063

**Published:** 2026-07-06

**Authors:** Rita Géczi, László Tóth

**Affiliations:** 1 School of Doctoral Studies, Hungarian University of Sports Science, Budapest, Hungary; 2 Department of Psychology and Sports Psychology, Hungarian University of Sports Science, Budapest, Hungary; 3 Teacher Training Institute, Hungarian University of Sports Science, Budapest, Hungary; 4 Institute of Health Promotion and Sport Sciences, Faculty of Education and Psychology, Eötvös Loránd University, Budapest, Hungary

**Keywords:** athletic development, longitudinal monitoring, physical performance, strength and conditioning, talent development, youth tennis

## Abstract

**Background:**

Athlete monitoring is considered an important component of youth athlete development and talent pathway management; however, ecologically valid longitudinal evidence within national tennis development systems remains limited.

**Purpose:**

Therefore, the present study examined temporal trends in physical performance among junior tennis players within a national talent development system.

**Methods:**

This retrospective repeated-measures monitoring study included 127 testing observations from 67 junior tennis players collected between 2021 and 2025. The testing battery assessed sprint speed, multidirectional movement, lower- and upper-body power, flexibility, and muscular endurance. Linear mixed-effects models were conducted for each performance variable, with testing year, chronological age, and sex included as fixed effects and athlete ID as a random intercept. Sensitivity analyses were performed using a repeated-athlete subsample and by including national ranking position as an additional covariate.

**Results:**

Significant year effects were observed for sprint performance and plank endurance. Sprint performance was fastest in 2021 and significantly slower in 2025, with similar findings in sensitivity analyses. The sprint difference between 2021 and 2025 (∼0.24 s) suggests a potentially meaningful difference in performance; however, its practical significance within junior tennis remains to be determined. Standing long jump and flexibility did not demonstrate statistically significant year effects, although both variables approached statistical significance. In contrast, multidirectional movement, push-up performance, and medicine ball throw performance remained relatively stable across testing years.

**Conclusions:**

Physical performance trends in junior tennis appeared to be non-linear and capacity-specific rather than uniform across performance domains. These findings highlight the value of multi-year monitoring within national talent development systems for identifying developmental variability and informing individualized and maturation-sensitive athlete development strategies in junior tennis.

## Introduction

Tennis is a complex, intermittent sport characterized by short, high-intensity rallies interspersed with relatively low effective playing time, yet involving substantial movement demands, frequent accelerations, and high stroke velocities (1). The physiological and mechanical loads experienced during both training and match-play require a high level of physical preparedness, particularly in terms of aerobic and anaerobic capacities, as well as speed and power (2, 3). Furthermore, congested competition schedules, such as playing multiple matches within a single day, may induce considerable fatigue and impair performance, highlighting the importance of appropriate recovery and load management strategies (4).

However, tennis performance is not determined solely by physical attributes, but rather by the complex interaction of technical, tactical, and psychological factors (5). Nevertheless, physical fitness remains a fundamental component, providing the basis for sport-specific performance and enabling the monitoring of individual performance trends (6). In this context, sport-specific, on-court testing procedures are particularly valuable, as they better reflect the actual movement patterns and demands of tennis compared to laboratory-based assessments (7).

The development of youth tennis players is strongly influenced by biological maturation, which may result in substantial inter-individual differences among athletes of the same chronological age (8). Early-maturing athletes often demonstrate superior physical performance, particularly in strength and speed-related capacities (9). To address these discrepancies, the concept of bio-banding has been proposed, grouping athletes based on biological maturity rather than chronological age (10). In addition, age- and sex-specific normative data and percentile systems may support a more accurate evaluation of youth athletes’ development (11). Recent longitudinal talent development research further suggests that physical performance trajectories may differ substantially according to maturity status, with developmental instability frequently observed around peak height velocity (12).

Longitudinal studies indicate that physical fitness in elite junior tennis players improves significantly with age, particularly between U14 and U16 (13). However, developmental patterns appear to differ between sexes: in boys, performance is more closely associated with biological maturation, whereas in girls, it is more strongly related to competitive level. Similarly, talent development trajectories may vary, with girls typically showing more linear improvements, while boys often exhibit delayed but accelerated development during adolescence (14). The predictability of performance increases with age, particularly during adolescence, which has important implications for talent identification.

The relationship between physical fitness and competitive performance is similarly complex. Although movement speed is considered a key determinant of tennis performance, its association with ranking is generally modest. Among its components, change of direction speed appears to have the strongest relationship with performance, largely influenced by lower-body power and linear speed (15).

Furthermore, elite player development pathways are shaped by the coordinated efforts of multiple stakeholders, including national federations, clubs, and coaches, whose roles vary across different stages of development (16). Long-term athlete development models, such as LTAD, further emphasize the importance of structured and stage-specific development frameworks (17, 18). Contemporary talent development literature increasingly emphasizes that athlete progression should be interpreted within long-term, individualized developmental frameworks rather than short-term performance outcomes alone (19).

Despite the growing body of literature examining physical performance in youth tennis, limited evidence is available regarding long-term physical performance trends within real-world national talent development systems. In particular, ecologically valid monitoring data collected across multiple years within federation environments remain scarce. Recent longitudinal athlete development studies using linear mixed-effects modelling have highlighted the value of repeated performance monitoring for capturing individualized temporal trends in youth sport environments (20).

Despite the recognized value of longitudinal athlete monitoring, ecologically valid multi-year datasets collected within national talent development systems remain relatively rare in the literature. This limitation has also been highlighted in talent identification research, where systematic reviews have emphasized the shortage of longitudinal evidence capable of evaluating long-term athlete development processes (21). Existing research in junior tennis has provided important insight into physical development during specific developmental periods and within selected cohorts, yet few studies have examined repeated physical performance assessments across multiple years within an operational national pathway (13, 22). Consequently, limited evidence is available regarding how physical performance characteristics evolve within real-world federation environments characterized by athlete turnover, changing cohort composition, and ongoing talent selection processes.

The present study addresses this gap by examining five consecutive years (2021–2025) of physical performance monitoring data collected within the Hungarian national junior tennis talent development system. To our knowledge, this is one of the first studies to describe multi-year physical performance trends using repeated monitoring data collected within a national junior tennis talent development system under real-world conditions.

## Methods

### Participants

This retrospective repeated-measures monitoring study included data collected from junior tennis players participating in the annual physical testing program of the Hungarian Tennis Federation between 2021 and 2025. A total of 127 testing observations from 67 individual athletes were included in the final analysis. Twenty-seven athletes contributed a single assessment, whereas 40 athletes participated in repeated assessments across multiple testing years. Specifically, 24 athletes were assessed twice, 13 three times, 2 four times, and 1 athlete participated in all five annual testing sessions (Table 1). This structure allowed for partial longitudinal tracking of athlete development while reflecting the natural athlete turnover within the national talent development system.

**Table 1:** Distribution of repeated assessments across athletes.

Number of assessments	Athletes (*n*)
1	27
2	24
3	13
4	2
5	1
Total	67

Across all testing years, the dataset included 65 observations from male athletes and 62 observations from female athletes representing multiple junior age categories (U12–U18). Participants were selected from the Hungarian junior talent development system, which primarily includes athletes ranked among the top performers in their respective age categories. These players were regularly invited to federation training camps and annual testing sessions. Additional athletes were occasionally included based on transitional selection decisions, recent competitive performance, or developmental considerations.

One observation was excluded from the final dataset due to injury-related performance limitations at the time of testing.

### Study design and procedures

The study employed a retrospective repeated-measures monitoring design using archival performance data collected as part of the annual athlete monitoring system of the Hungarian Tennis Federation. Physical assessments were conducted once per year between 2021 and 2025 at the National Training Center under standardized conditions. The same testing protocols, equipment, and standardized assessment procedures were applied across all testing years. Testing was conducted by experienced strength and conditioning professionals affiliated with the Hungarian Tennis Federation. Prior to testing, athletes completed a standardized 15–20-min warm-up supervised by certified strength and conditioning professionals. Although repeated measurements were available for a substantial proportion of athletes, the dataset did not represent a fully closed longitudinal cohort due to athlete turnover across testing years.

The retrospective use of anonymized performance monitoring data was approved by the Research Ethics Committee of the Hungarian University of Sports Science (approval number: MTSE-KEB/No21/2025). Data collection procedures were conducted within the annual athlete monitoring system of the Hungarian Tennis Federation.

Importantly, the present dataset reflects a real-world athlete monitoring environment characterized by natural athlete turnover and evolving cohort composition within the national development pathway. Consequently, year effects should be interpreted as temporal monitoring trends rather than definitive longitudinal developmental trajectories. To evaluate the robustness of significant findings, sensitivity analyses were conducted using only repeatedly assessed athletes.

### Physical performance measures

Before testing, athletes completed a standardized 15–20-min warm-up consisting of dynamic mobility exercises, running drills, and sport-specific movement preparation.

Anthropometric measurements. Body height was measured to the nearest 0.1 cm using a stadiometer, while body mass was recorded to the nearest 0.1 kg using a calibrated digital scale.

All assessments were administered according to the official Hungarian Tennis Federation testing protocol. The testing battery included anthropometric and performance-based assessments targeting speed, agility, lower-body power, upper-body power, flexibility, core endurance, muscular endurance, and aerobic fitness.

#### 20-m sprint test

Linear sprint performance was assessed over 20 m using OXA Sport electronic timing gates (OXA Sport, Hungary). Athletes performed one familiarization trial followed by three maximal trials from a standing start position, with one-minute passive recovery between attempts. The fastest trial was retained for analysis.

#### Agility test

Tennis-specific agility was assessed using a lateral movement task involving the relocation of tennis balls between cones positioned 4 m apart. Athletes completed one familiarization trial followed by three recorded trials, and the best result was used for analysis.

#### Hexagon test

Movement coordination was evaluated using the hexagon jump test. Players completed one familiarization trial followed by two maximal trials, with the best performance retained.

#### Standing long jump

Lower-body explosive power was assessed using a bilateral standing long jump. Following one practice attempt, athletes performed repeated maximal trials until no further improvement was achieved, and the best result was recorded.

#### Star run test

Tennis-specific multidirectional movement speed was assessed using a star-shaped running task where players collected five tennis balls from predefined court locations. Two valid trials were completed, and the better result was retained.

#### Sit-and-reach test

Hamstring and lower-back flexibility were assessed using a standardized sit-and-reach protocol. One valid attempt was recorded.

#### Plank test

Core endurance was evaluated using an isometric forearm plank hold. Time was recorded until technical failure.

#### Push-up test

Upper-body muscular endurance was assessed by recording the maximum number of technically correct push-ups completed within 30 s.

#### Medicine ball throws

Tennis-specific upper-body power was assessed using both forehand and backhand medicine ball throws. U12 and U14 athletes used a 2-kg medicine ball, whereas U16 and U18 athletes used a 3-kg ball. Athletes performed repeated attempts until no further improvement was achieved, and the best performance was retained. Because chronological age was included as a fixed effect in all mixed-effects models, age-related differences associated with medicine ball mass were partially controlled statistically. Nevertheless, results for medicine ball performance should be interpreted with caution, and the potential influence of different ball masses is acknowledged as a study limitation.

#### 20-m shuttle run test

Aerobic capacity was assessed using the multistage 20-m shuttle run test according to standardized procedures. Performance was recorded as the final successfully completed stage

Although the 20-m shuttle run and hexagon tests were included in the original testing battery, these variables were excluded from longitudinal analyses due to inconsistent administration across testing years and substantial missing data, which prevented reliable estimation within the mixed-effects modelling framework.

## Statistical analysis

Descriptive statistics (mean ± standard deviation, minimum, maximum, and valid sample size) were calculated for all anthropometric and physical performance variables across each testing year (2021–2025). Sex distribution across testing years was examined using contingency table analysis.

To evaluate temporal trends in physical performance across the five-year period, separate linear mixed-effects models were conducted for each physical performance variable. In each model, the performance outcome served as the dependent variable, while testing year, chronological age, and sex were entered as fixed effects. Testing year was modeled as a categorical fixed effect, whereas chronological age was included as a continuous covariate. Athlete identity was included as a random intercept to account for repeated observations from athletes who participated in multiple annual assessments. Models were estimated using restricted maximum likelihood (REML) with Satterthwaite approximation for degrees of freedom.

This modeling approach allowed for appropriate handling of the unbalanced longitudinal structure of the dataset and provided robust estimates of temporal effects.

Estimated marginal means with 95% confidence intervals were calculated for each testing year to examine adjusted yearly trends. Statistical significance was set at *p* < 0.05 (two-tailed). All analyses were performed using JASP statistical software (Version 0.19.3.0; University of Amsterdam, Amsterdam, the Netherlands).

To further evaluate the robustness of significant findings, a sensitivity analysis was conducted including only athletes who participated in at least two testing occasions (*n* = 40). This subsample comprised observations from the repeatedly assessed athlete group. Separate linear mixed-effects models were repeated for 20-m sprint and plank performance, as these variables demonstrated significant year effects in the primary analyses. Variables without significant year effects in the primary models were not included in the sensitivity analyses. National ranking position at the time of physical testing was also included as an additional covariate in selected models to examine whether annual differences in competitive level influenced observed temporal trends. Lower numerical values indicated higher competitive ranking positions.

Descriptive characteristics of the sample across testing years are presented in Table 2.

**Table 2:** Descriptive characteristics of Hungarian junior tennis players across testing years (2021–2025).

Year	*n*	Male	Female	Age (years)	Height (cm)	Body mass (kg)
2021	20	16	4	12.62 ± 2.24	160.43 ± 17.22	49.61 ± 14.23
2022	15	5	10	13.02 ± 1.38	164.70 ± 10.17	53.61 ± 11.20
2023	23	6	17	14.10 ± 2.21	167.89 ± 14.65	57.61 ± 14.53
2024	32	19	13	13.91 ± 1.83	165.03 ± 12.22	54.32 ± 13.70
2025	37	19	18	13.89 ± 2.04	165.33 ± 12.25	54.71 ± 14.88

To examine longitudinal changes in physical performance, linear mixed models were conducted for each fitness variable, controlling for chronological age and sex, with athlete ID included as a random effect.

The results of the linear mixed-effects models are summarized in Table 3.

**Table 3:** Fixed effects from linear mixed-effects models examining temporal trends in physical performance variables across the five-year period.

Variable	Year (*F*)	Year (*p*)	Age (*F*)	Age (*p*)	Sex (*F*)	Sex (*p*)
20-m sprint	8.01	<.001	100.71	<.001	4.47	.038
Star run	1.25	.297	73.65	<.001	4.40	.039
Standing long jump	2.26	.070	125.04	<.001	5.70	.020
Plank	2.61	.041	3.98	.049	1.44	.235
Flexibility	2.47	.052	11.68	.001	5.10	.027
Push-up	0.71	.589	6.61	.012	11.92	<.001
Medicine ball forehand throw	0.59	.672	121.34	<.001	18.71	<.001
Medicine ball backhand throw	1.27	.288	137.38	<.001	15.15	<.001

**Figure 1:**
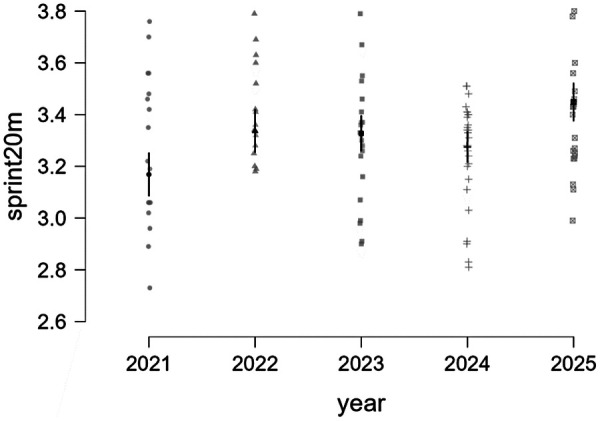
Estimated marginal means (±95% confidence intervals) for 20-m sprint performance across testing years among repeatedly assessed athletes (*n* = 40).

A significant effect of testing year was observed for 20-m sprint performance (*F* = 8.01, *p* < .001), indicating meaningful differences across annual cohorts. Estimated marginal means demonstrated that sprint performance was fastest in 2021 (3.19 s) and slowest in 2025 (3.44 s), with non-linear fluctuations across intermediate years. Planned contrast analysis further revealed that athletes tested in 2025 performed significantly slower than those assessed in 2021 (Δ = 0.24 s, *p* < .001). The magnitude of this difference [∼0.24 s; 95% CI (−0.34, −0.15)] suggests a potentially meaningful difference in sprint performance between annual cohorts; however, the practical significance of this difference within junior tennis remains to be determined. Estimated marginal means from the repeated-measures sensitivity analysis are presented in Figure 1.

**Figure 2:**
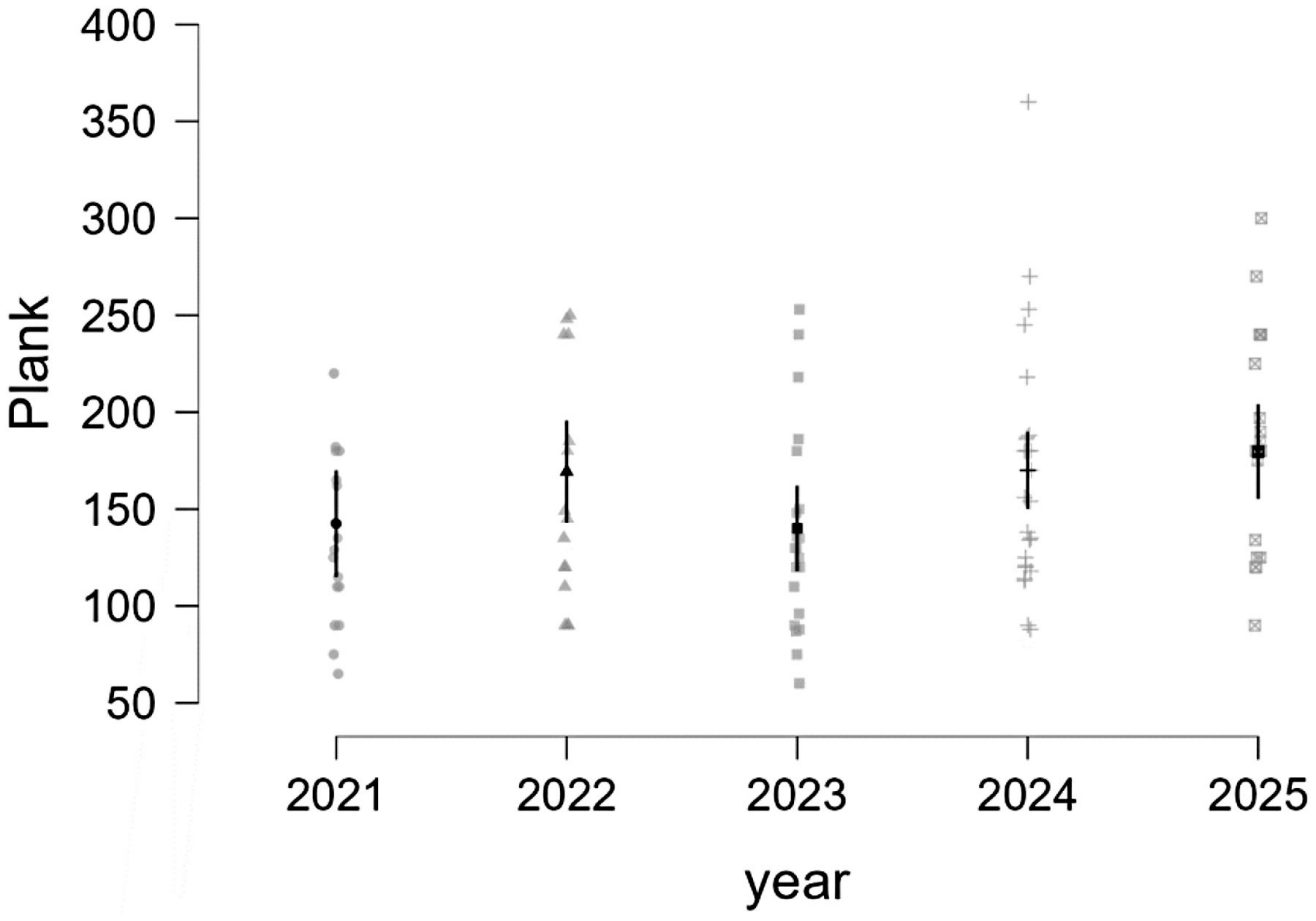
Estimated marginal means (±95% confidence intervals) for plank endurance across testing years among repeatedly assessed athletes (*n* = 40).

A significant year effect was observed for plank performance (*F* = 2.61, *p* = .041), indicating temporal fluctuations in core endurance across testing years. Estimated marginal means from the repeated-measures sensitivity analysis are presented in Figure 2.

No significant year effects were observed for star run agility, push-up performance, medicine ball forehand throw, or medicine ball backhand throw. Flexibility (*p* = 0.052) and standing long jump (*p* = 0.070) did not demonstrate statistically significant year effects, although both variables approached statistical significance.

A supplementary sensitivity analysis including only repeatedly assessed athletes (*n* = 40) yielded comparable findings. The significant year effect for 20-m sprint performance remained significant (*F* = 7.68, *p* < .001), with athletes assessed in 2021 demonstrating the fastest sprint times (3.17 s) and those assessed in 2025 demonstrating the slowest sprint times (3.45 s). Similarly, the year effect for plank endurance remained significant (*F* = 2.83, *p* = .030), indicating that the observed temporal trends were not solely driven by athlete turnover.

Importantly, the temporal sprint effect remained significant after controlling for national ranking position, suggesting that the observed differences were not solely attributable to competitive level variation across annual cohorts.

Chronological age was a significant predictor in nearly all models, with increasing age generally associated with improved physical performance outcomes. Sex differences were identified across several physical performance variables, although the magnitude and direction of these effects varied across tests.

## Discussion

The present study examined five-year trends in the physical performance characteristics of Hungarian junior tennis players within a national talent development system. The primary finding was that temporal performance trends appeared to be non-linear, capacity-specific, and potentially influenced by broader contextual and cohort-related factors. Significant differences across testing years were identified in linear sprint performance and core endurance, whereas lower-body power did not demonstrate statistically significant variation across testing years. To our knowledge, this study is among the first studies to describe multi-year physical performance monitoring trends within a national junior tennis talent development system.

Linear mixed-effects modelling has recently been recommended for longitudinal athlete monitoring datasets characterized by repeated measures, athlete turnover, and unbalanced cohort structures (20).

One of the most notable findings of the present study was the significant variation observed in 20-m sprint performance across testing years. Sprint performance was fastest in 2021 and significantly slower in 2025, with non-linear fluctuations across intermediate years. Importantly, this temporal effect remained significant in both the repeated-athlete sensitivity analysis and models controlling for national ranking position, supporting the robustness of the finding. Temporal variation may additionally reflect evolving selection dynamics and cohort composition within the national talent development system.

Although the mechanisms underlying these temporal differences cannot be directly determined, the consistency of the findings across sensitivity analyses suggests that the observed variation may reflect a combination of cohort-related and developmental influences rather than random fluctuation alone. However, the present dataset did not include direct measures of biological maturation, training exposure, or neuromuscular training content, despite consistent testing procedures across years. These factors are known to substantially influence sprint development during adolescence.

Sprint performance during adolescence is particularly sensitive to growth-related changes in muscle mass, coordination, and force production, as well as to the quality and consistency of speed-oriented training stimuli.

From an applied perspective, these findings highlight the importance of continuous monitoring of linear speed within junior tennis development programs. Rather than assuming linear improvement across age groups, practitioners should consider the possibility of non-linear and capacity-specific development patterns, particularly during periods of rapid growth and maturation.

Given that linear speed is considered an important component of tennis movement performance, the observed differences across testing years highlight the value of continued monitoring within national talent development systems.

Lower-body explosive power demonstrated modest fluctuations across testing years; however, these differences did not reach statistical significance. Explosive performance during adolescence is strongly influenced by maturation-related neuromuscular adaptations, which may contribute to inter-individual variability in temporal trends. These findings further support the need to interpret physical testing outcomes within an individualized developmental framework rather than relying solely on chronological age.

In contrast, agility and upper-body power remained relatively stable across testing years. This finding may indicate less pronounced temporal variation in these capacities; however, the underlying reasons cannot be determined from the present dataset.

Chronological age significantly influenced nearly all physical performance outcomes, which aligns with previous literature demonstrating substantial developmental changes during adolescence.

Sex differences were also identified across several physical variables, supporting previous research showing divergent developmental pathways between male and female youth athletes during adolescence.

Collectively, these findings highlight the importance of individualized monitoring systems in youth tennis and further support the importance of maturation-sensitive evaluation frameworks within youth athlete development environments. Taken together, the results emphasize that temporal performance trends in junior athletes are non-linear and capacity-specific, underscoring the need for longitudinal, context-sensitive evaluation frameworks.

### Practical implications

From an applied perspective, this study provides one of the first multi-year physical performance monitoring datasets within Hungarian junior tennis. These findings may contribute to future efforts aimed at establishing sport-specific normative benchmarks, identifying developmental plateaus, monitoring long-term trends, and refining evidence-based talent identification strategies. Practitioners should avoid assuming linear year-to-year physical development during adolescence and instead implement individualized, maturation-sensitive monitoring approaches capable of identifying developmental variability and temporary performance plateaus.

Furthermore, integrating biological maturation indicators into future monitoring systems may improve individual athlete evaluation and reduce premature selection biases. More consistent longitudinal assessment of aerobic fitness and age-appropriate coordination measures may further strengthen long-term athlete monitoring systems.

### Limitations

Several limitations should be acknowledged.

First, the dataset did not represent a fully closed longitudinal cohort, as not all athletes participated in every testing year. Furthermore, sex distribution differed significantly across testing years, which may have contributed to some observed year effects despite statistical adjustment for sex within the mixed-effects models.

Second, biological maturation indicators were not assessed, limiting the interpretation of developmental differences. Additionally, training volume, competition exposure, and sport-specific practice characteristics were not available, limiting causal interpretation of the observed performance trends. This may be particularly relevant given recent longitudinal evidence demonstrating maturity-specific temporal trends in youth athletes (12).

Third, technical, tactical, psychological, and competitive performance variables were not included.

Although ranking position was included in supplementary analyses, ranking may not fully capture competitive performance quality due to differences in tournament exposure and scheduling opportunities.

In addition, different medicine ball masses were used across age categories, which may have influenced upper-body power outcomes despite statistical adjustment for chronological age.

Finally, the findings reflect the Hungarian national tennis development system and may not be fully generalizable to other countries or competitive structures.

Consequently, observed year effects should be interpreted as temporal monitoring trends rather than direct evidence of longitudinal developmental trajectories.

## Conclusion

This study examined five-year trends in physical performance among junior tennis players within a national talent development system, providing one of the first multi-year monitoring datasets in this context. The findings indicate that temporal performance trends are non-linear and capacity-specific, with significant temporal variation observed in sprint performance and core endurance, while other capacities remained relatively stable.

Importantly, these findings suggest that changes in physical performance across annual cohorts may not follow uniform patterns and are likely influenced by a combination of developmental and contextual factors.

Collectively, these findings support the integration of longitudinal and ecologically valid athlete monitoring systems within national tennis development environments to better capture developmental variability and support individualized decision-making processes.

## Data Availability

The raw data supporting the conclusions of this article will be made available by the authors, without undue reservation.
